# The Pivotal Immunoregulatory Functions of Microglia and Macrophages in Glioma Pathogenesis and Therapy

**DOI:** 10.1155/2022/8903482

**Published:** 2022-04-04

**Authors:** Seidu A. Richard

**Affiliations:** Department of Medicine, Princefield University, P. O. Box MA 128, Ho, Ghana

## Abstract

Gliomas are mixed solid tumors composed of both neoplastic and nonneoplastic cells. In glioma microenvironment, the most common nonneoplastic and infiltrating cells are macrophages and microglia. Microglia are the exact phagocytes of the central nervous system, whereas macrophages are myeloid immune cells that are depicted with ardent phagocytosis. Microglia are heterogeneously located in almost all nonoverlapping sections of the brain as well as the spinal cord, while macrophages are derived from circulating monocytes. Microglia and macrophages utilize a variety of receptors for the detection of molecules, particles, and cells that they engulf. Both microglia and peripheral macrophages interact directly with vessels both in the periphery of and within the tumor. In glioma milieu, normal human astrocytes, glioma cells, and microglia all exhibited the ability of phagocytosing glioma cells and precisely apoptotic tumor cells. Also, microglia and macrophages are robustly triggered by the glioma via the expression of chemoattractants such as monocyte chemoattractant protein, stromal-derived factor-1, and macrophage-colony stimulating factor. Glioma-associated microglia and/or macrophages positively correlated with glioma invasiveness, immunosuppression, and patients' poor outcome, making these cells a suitable target for immunotherapeutic schemes.

## 1. Introduction

Gliomas are diverse solid tumors composed of both neoplastic and nonneoplastic cells [1]. In glioma milieu, the most common nonneoplastic and infiltrating cells are macrophages and microglia [2–4]. In the malignant glioma milieu, both resident microglia and macrophages derived from circulating monocytes form the glioma-infiltrating immune cells and are the key contributors to glioma progression [5, 6]. Microglial cells usually are the participant in the early stage, while the macrophages derived from circulating monocytes are the key participant in the later stage to promote glioma growth [5, 6].

Microglia and macrophages are vigorously triggered by the glioma via the expression of chemoattractants such as monocyte chemoattractant protein (MCP-1, also known as CCL2), stromal-derived factor-1 (SDF-1), and macrophage-colony stimulating factor (M-CSF) [7, 8]. A study revealed that glioma-associated microglia and/or macrophages (GAMs) exhibit significant diversity as well as plasticity and show a partially known distinctive phenotype, only partly ascribable to inflammatory (M1) or alternative (M2) polarization secretory forms [9]. Circadian rhythm is a known phenomenon that regulate and maintain homeostasis in normal cells as well as tissues [10–12].

Studies have demonstrated that it stimulates cancer-relevant processes like cell proliferation and survival, DNA repair, metabolism, and inflammation [11, 12]. Circadian locomotor output cycles kaput (CLOCK) and brain and muscle ARNT-like 1 (BMAL1) also identified as aryl hydrocarbon receptor nuclear translocator-like protein 1 (ARNTL) are two fundamental transcription factors of the circadian mechanism, which comprise of a heterodimeric complex [10, 13]. This complex was capable of triggering the secretion of the period (PER) and cryptochrome (CRY) genes, which eventually constituted a negative feedback loop that blocked the activity of CLOCK : BMAL1 complex [10, 13]. Microglia and macrophages are key determinants of this mechanisms [10, 13].

This review focuses on the pivotal role of microglia and macrophages in glioma pathogenesis as well as therapy. The “Boolean logic” was utilized to search for article on the subject matter. Most of the articles were indexed in PubMed with strict inclusion criteria being the role of microglia and macrophages at the glioma microenvironment. The search terms were microglia and macrophages and/or glioma.

## 2. Microglia

**Figure 1:**
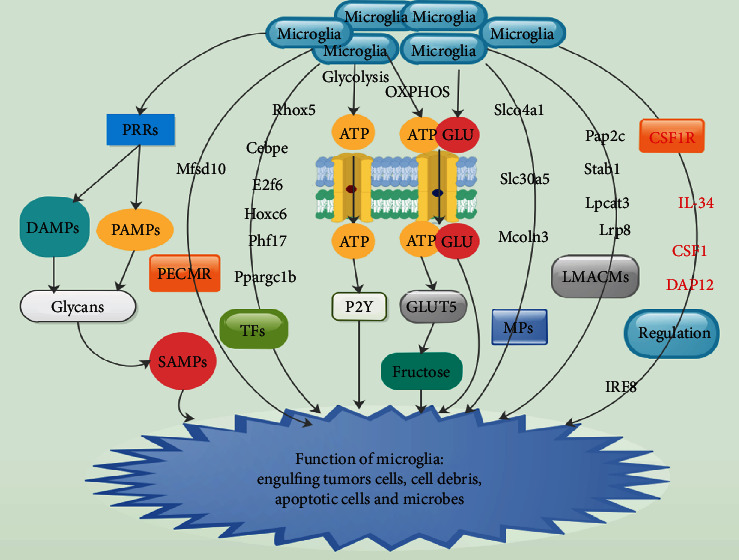
Function of microglia in the central nervous system. Red factors = inhibitor/inhibition; black factors = facilitator/facilitation.

Microglia are the precise phagocytes of the central nervous system (CNS) [14, 15]. They are heterogeneously located in almost all nonoverlapping sections of the brain as well as the spinal cord [14–16]. Functionally, they are capable of detecting as well as engulfing extracellular material such as tumors cells, cell debris, apoptotic cells, and microbes (Figure 1) [15]. Thus, they provide significant support to the function as well as structure of the CNS [15]. Studies have demonstrated that microglial cells comprehensively communicate with neuronal circuits in developing as well as in the adult brain [16–18]. Furthermore, microglia triggered neuronal apoptosis, eradicated less active synaptic connections such as synaptic pruning, and stimulated neuronal activity [19–21].

Also, studies revealed that microglia stimulated synapse formation in the mature brain [18, 22, 23]. Microglia form a 3-dimensional network in the CNS, and they communicate via hemichannels as well as gap junctions (Figure 1) [15, 24, 25]. Studies have shown that the accumulation of microglial at a pathological area is initiated by “danger signals,” such as extracellular ATP as well as its derivatives, which target purinoreceptors of the adenosine diphosphate receptor (P2Y) family (Figure 1) [26, 27]. The hemichannels permit expression as well as uptake of glutamate (GLU) and ATP which are necessary initiates of communication between neurons and astrocytes (Figure 1) [15]. The gap junctions also permit microglia to function as a syncytium [15]. Nevertheless, the significance and the level of these interconnections need further studies.

Transcriptome study on the mouse microglia showed distinctive qualities of recently isolated brain-derived cells, while the cultured cells showed characteristic of stimulated microglia [15, 28]. Transcription factors (TFs) such as Rhox5, Cebpe, E2f6, Hoxc6, Phf17, and Ppargc1b are expressed by microglia (Figure 1) [15, 28]. Furthermore, several membrane proteins (MPs) such as the ion transporters Slco4a1, Slc30a5, and Mcoln3 have been recognized in microglia that are distinctive and not secreted by other macrophages (Figure 1) [15, 28]. Also, the lipid metabolism associated cell membrane molecules (LMACMs) Lrp8, Lpcat3, Stab1, and Pap2c, and the putative efflux cell membrane receptor (PECMR) Mfsd10 is expressed by microglia (Figure 1) [15, 28].

Resident microglia secrets pattern recognition receptors (PRRs), which sense pathogen-associated molecular patterns (PAMPs) like microbial pathogens as well as damage-associated molecular patterns (DAMPs) such as the adenine nucleotides (ATP/ADP) (Figure 1) [16, 29].

RodrÍguez et al. demonstrated that PAMPs and DAMPs are influenced by glycans recognized as self-associated molecular patterns (SAMPs), which act as modifiers in tumor cells, blocking immune response in their milieus (Figure 1) [29]. They further indicated that the utilization of glycans by cancer cells facilitates immune suppression by regulating the differentiation of GAMs [29].

Ghosh et al. revealed that microglia were capable of producing ATP via glycolysis as well as oxidative phosphorylation (OXPHOS), highly secreted glucose transporter (GLUT)-5, which had an extreme affinity for fructose (Figure 1) [30]. It was established that microglial population is regulated via signals originating from the binding of colony stimulating factor 1 (CSF1) as well as interleukin- (IL-) 34 to the microglial CSF1 receptor (CSF1R) (Figure 1) [31–33]. Furthermore, mice defective of CSF1R or IL-34 or the CSF1R adaptor protein DNAX activation protein of 12 kDa (DAP12) had significantly decreased quantities of tissue macrophages as well as microglia (Figure 1) [31–33]. Transcription factor, interferon regulatory factor- (IRF-) 8, was responsible for the development of microglia because, IRF8-deficient mice exhibited expressively decreased microglia concentration in adults (Figure 1) [31, 34].

## 3. Anatomical Localization of CNS Macrophages

In the CNS, macrophages are categorized into perivascular macrophages, meningeal macrophages, macrophages of the circumventricular organs, and macrophages of the choroid plexus according to their anatomical locations [35]. Studies have shown that all CNS macrophages located in the perivascular or Virchow-Robin spaces, subdural meninges, and choroid plexus originated from short-lived blood monocytes after birth which often rapidly substituted by bone marrow- (BM-) derived cells [35–37].

Studies have further demonstrated that perivascular and meningeal macrophages are produced from embryonic yolk sac precursors, while choroid plexus macrophages have dual embryonic as well as adult hematopoietic origins [35, 38, 39]. A study established that CNS are often restricted at the interface between the parenchyma and the circulation [39]. Studies have demonstrated that perivascular macrophages exist in the CNS parenchyma after BM transplantation in chemotherapeutical conditioning milieu [40, 41]. CNS macrophages comprise of a complex network of heterogeneous cell populations, and together with other blood-borne myeloid cells that infiltrate the brain under certain conditions, macrophages may potentially lead to novel therapies for brain diseases [35, 38, 39].

BM chimeras using irradiation of the recipient exhibited that macrophages at CNS boundaries originated from blood-borne myeloid cells during adulthood [42, 43]. Goldmann et al. demonstrated that transplantation of BM from Acta1-GFP mice resulted in robust dissemination of donor-derived GFP^+^Iba-1^+^ macrophages in the subdural meninges, perivascular spaces, and choroid plexus, while microglial switch was restricted [35]. Kvisten et al. investigated the histopathological aspects of GAMs in human GBMs with emphasis on the number, distribution, and morphology of Iba1- and CD68-immunoreactive GAMs, as well as the relationship with tumor growth estimated from magnetic resonance imaging scans [44].

## 4. Microglia and Macrophage Subtypes

Microglia exists in three distinct kinds which serve diverse functional roles in the CNS [45]. These three microglia forms are amoeboid, ramified, and reactive microglia [45]. The amoeboid microglia are linked to the embryonic CNS development [46, 47]. These cells composed of a round cell body, pseudopodia, and a thin filopodia-like processes [46, 47]. They contain several lysosomes and attributes suggestive of a motile phagocytic phenotype [46, 47]. These cells are seen late in conception and vanish quickly after birth in rats [46, 47]. These cells function as tissue histogenesis responsible for the elimination of inapt as well as unessential axons and also aid in the advancement of axonal migration as well as growth [48–50]. These cells change into ramified microglia in the adult CNS [51].

On the other hand, ramified microglia exist in copiousness quantities in the brain parenchyma and comprise of about 10-20% of the entire quantity of glial cells in the adult CNS [52]. These cells types are tin and round in nature consisting of copious branching processes with very minute cytoplasm [53]. These cells showed pinocytotic actions as well as confined motility and are preserved via local cell division as well as the recruitment of circulating peripheral blood monocytes [45, 54]. They contribute to metabolite removal as well as the elimination of toxic factors secreted from injured neurons and are capable of transforming into neurons, astrocytes, or oligodendrocytes [45, 55].

Reactive microglia are rod-like, with nonbranching processes and copious lysosomes as well as phagosomes [56, 57]. These reactive cells constitute a cluster of macrophages related to brain injury as well as neuroinflammation [56, 57]. Also, reactive microglia secrete MHC class II antigens as well as other surface molecules comprising of CD40, B7, and ICAM-1which are essential for antigen presentation [58, 59]. Furthermore, reactive microglia express inflammatory mediators, which coordinate the cerebral immune response [58, 59]. Moreover, reactive microglia often amass at the site of damage where they participate in neuroprotective function by phagocytosing injured cells as well as debris after an injurious event [60].

Microglial subtypes are also defined by differential gene expressions into keratan sulfate proteoglycan- (KSPG-) microglia, Hox8b-microglia, CD11c-microglia, TREM2-microglia, microglia-supporting neurogenesis, single-cell RNAseq data, and proliferative-region-associated microglia (PAM) [61]. On the other hand, M1 or classically activated macrophages were subgrouped in M1a and M1b [62], while M2 or alternative activated macrophages are subdivided into four distinct subtypes such as M2a, M2b, M2c, and M2d, depending on the type of inducing agent as well as the expressed markers [63]. It is worth noting that the association between amoeboid, ramified, and reactive microglia and glioma needs urgent investigation. Also, the association between M1 macrophages like M1a and M1b as well as M2 macrophages subtype like M2a, M2b, M2c, and M2d and glioma needs further investigation.

## 5. Differences between Microglia and Macrophages

Microglia, which are widespread in the CNS, originate from yolk sac progenitors and migrate to the brain during the early stages of development [14, 64]. They are the resident macrophages of the CNS and constitute about 20% of the entire quantity of cells in the CNS [14, 64]. Studies have shown that these cells are scattered in all regions of the brain, and their concentration differs according to the area, varying from about 5% in the corpus callosum to about 12% in the substantia nigra [14, 64–66]. These cells conserve CNS homeostasis by modulating synapses based on neuronal activity as well as the concurrent intercommunication between neurons and astrocytes [64, 65].

Studies have demonstrated that microglial cells come together during fetal development, childhood, and puberty resulting in “synaptic pruning” as well as the generation of neurotrophic factors [64, 67, 68]. Microglia share many functions with peripheral macrophages; polarized microglial populations can be differentiated from polarized macrophages by protein secretory profiles, phagocytic capability, and response to injuries [69, 70]. Moreover, M2 microglia appear to offer more protection than M2 macrophages *in vitro* and exhibit a higher affinity to conserve their M2 status [69, 70]. Studies have established that both microglia and bone marrow-derived macrophages in the CNS are associated with phenotype switching but may participate differently to CNS repair *in vivo* animal models [71–73].

Macrophages are myeloid immune cells that are depicted with fervent phagocytosis. It was speculated that macrophages are derived from circulating monocytes based on fact that, in pathological situations, monocytes produce macrophages [74, 75]. Monocytes, specifically the Ly6Chi monocytic subclass, exhibit a short half-life just like neutrophils [74, 76]. Thus, these cells compose of precursor reservoir for tissue-resident mononuclear phagocytes. Transplantation investigations exhibited near-total reorganization of tissue macrophage populations, with the exclusion of Langerhans cells as well as microglial cells [74, 77]. Microglia perform functions analogous to macrophages such as phagocytosis as well as antigen presentation [78]. They also perform extra functions in homeostasis like the expression of neurotrophic factors that are fundamental for both normal preservation and response to pathological disorders [78, 79].

Microglia are peripatetic within their own distinctive zones and totally scan the brain parenchyma countless times a day [78, 80]. Thus, they function as fundamental component to normal parenchymal immune surveillance [80]. Studies have demonstrated that microglia are sensitive to ATP, potassium, and purinoceptor inhibitors and are capable of sensing neuronal cell death as well as other pathological features with high acuity while scanning [81, 82]. Microglia are transformed into amoeboid phenotypes upon stimulation, and they act analogously like macrophages with extreme metabolic rate, quick migration to the lesion source, and expressing of IL-6, IL-1*β*, and tumor necrosis factor alpha (TNF*α*) before phagocytosing as required [78, 83].

RodrÍguez et al. indicated that the use of CD45 antibodies exhibited low secretory levels for resident microglia (CD45^low^) as well as high secretory levels for CNS macrophages (CD45^high^) [29]. Greter et al. established that leukocyte antigen CD45 was upregulated in activated microglia and thus could be used as a marker to differentiate microglia from blood-derived immigrated macrophage populations as well as blood monocytes which had a decreased secretion of the common leukocyte antigen CD45 [84]. Bowman et al. also demonstrated that CD49D was deficient in microglia and can be used to differentiate them from CNS macrophages in mouse as well as human brain tumors [85].

Studies have demonstrated that ionized calcium-binding adaptor molecule (Iba1) which is extremely preserved in mammals was an advantageous and specific marker for the recognition of microglia, since its detection [86, 87]. Several studies have further demonstrated that Iba1 is not secreted in blood monocytes, but often also in blood-derived tissue macrophages as well as dendritic cells [84, 88, 89]. Studies have demonstrated that similar microglia markers such as the major histocompatibility complex (MHC) class II, the fractalkine receptor (CX3CR1), and Sall1can be utilized to differentiate parenchymal microglia from border-associated macrophages [26, 90].

Macrophages are also recruited glioma milieu from peripheral hematopoietic stem cell compartments although microglia are the resident tissue macrophage of the brain [38, 91]. Microglia and macrophages originate from different location but exhibit similar functions in glioblastoma multiforme (GBM) [38, 91]. Thus, brain macrophages are also associated with brain homeostasis as well as immune responses in pathological states [38]. Nevertheless, in GBM, microglial cells and infiltrating macrophages amass within and around the tumor mass, but they are ineffective in fighting tumor development or can even strengthen the tumor [91].

## 6. Microglia and Macrophages Subtypes in GBM

GBM milieu often compose of both microglia and macrophages which both have classically been subdivided into M1 (proinflammatory) as well as M2 (immunosuppressive) phenotypes to distinguish them as either possessing antitumor or tumor-promoting (protumor) activities, correspondingly [78]. Mills et al. were the first to propose the M1/M2 dichotomy as a way to differentiating the phenotypic preferences of macrophages from the perspective of T helper (Th)1 as well as Th2 lineages in CD4^+^ T cells [92]. They indicated that M1 denoted macrophages with Th1 linages which generated inflammatory stimulated nitric-oxide species (iNOS), whereas M2 denoted macrophages with Th2 which generated more cell division-inducing polyamines, like ornithine [92].

It was established that macrophages can be polarized into M1 or M2 phenotype depending on the stimulus [7, 93]. M1 macrophages are referred to as classically activated, or proinflammatory macrophages, and are induced in response to inflammatory stimuli such as lipopolysaccharides (LPS), interferon- (IFN-) *γ*, and GM-CSF [7, 93]. Furthermore, M1 macrophages express proinflammatory cytokines like TNF*α*, IL-6, and CXCL10, present antigen to immune cells and phagocytize tumor cells [7, 93]. M2 macrophages are referred to as alternatively induced, or immunosuppressive, and are stimulated in response to stimuli such as IL-4, IL-13, and M-CSF [7, 93]. Moreover, M2 macrophages express immune-suppressive cytokines like IL-10 as well as transforming growth factor-beta (TGF-*β*), promote T regulatory (Treg) cell differentiation, and assist in tumor progression. These macrophage features are analogous to microglia [7, 93].

Studies have demonstrated that the alternative macrophage activation had subdivisions like M2a which is responsible for Th2 responses, type II inflammation, killing of pathogens, and allergy, M2b which is also responsible for Th2 activation as well as immunoregulation, and M2c which is responsible for immunoregulation, matrix deposition, and tissue remodeling [94–96]. These polarized subpopulations of macrophages vary in terms of receptor secretion, effector function, cytokine, and chemokine generation [94, 97]. Studies revealed that microglial and macrophage populations are characterized by CD11b^+^/CD45^dim^ (microglia) and CD11b^+^/CD45^high^ (macrophages) phenotypes and constitutes about 13-34% (microglia) as well as about 4.2-12% (macrophages) of the tumor cell mass in experimental gliomas [98, 99].

## 7. Glioma-Associated Microglia and/or Macrophages at Glioma Microenvironment

**Figure 2:**
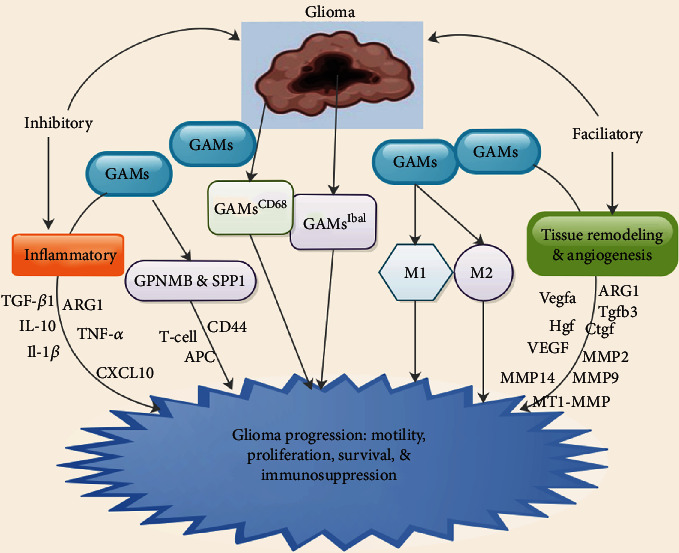
Influence of glioma-associated microglia and/or macrophages (GAMs) at the glioma microenvironment.

Majority of the nonneoplastic cells in glioma are GAMs either of peripheral origin or brain resident microglia [1, 94, 100]. GAMs constitute about 30% of the entire glioma mass and partakes in numerous functions in GBM progression such as motility, proliferation, survival, and immunosuppression (Figure 2) [1, 94, 100]. Furthermore, the GAM structure consists of a collection of differentiation 15% of CD11b^+^/CD45^dim^-activated resident microglia which are mostly localized in peritumoral zones, as well as 85% CD11b^+^/CD45^high^-infiltrative peripheral derived monocyte macrophages which are mainly localized in perivascular zones [101].

Kvisten et al. observed that, in specific glioma regions, there were more GAMs^CD68^ in the slow-growing GBMs, whereas the quantities of GAMs^Iba1^ were analogous similar to slow- and fast-growing gliomas [44]. There were expressively more GAMs^Iba1^ compared to GAMs^CD68^ (Figure 2) [44]. It was revealed that the lba1 antibody interacts with an ionized calcium-binding protein characteristic for both resting and stimulated microglia/macrophages, while anti-CD68 label lysosomal membranes are detected in these cells [102]. It was further observed that phenotypes and activation states of GAMs were more relevant [102]. Moreover, ramified GAMs were more predominate in the peripheral parts of the tumor compared to the central parts where GAMs predominantly had amoeboid phenotypes [44].

Studies have demonstrated that most GAMs were situated in perivascular regions and were best envisaged in CD68 stained sections, particularly in the infiltration zone [103–105]. This reflected the existence of GAMs in perivascular niches, in which there was an association between different cell types [103–105]. Studies further revealed that GAMs were mostly diffusely disseminated although they concentrated in microanatomical compartments coherent with perivascular as well as per necrotic niches [104, 106]. Hambardzumyan et al. observed that the blood-brain barrier was frequently compromised resulting in an infiltration of peripheral macrophages in CNS diseases [94].

Studies have demonstrated that GAMs display a mixed M1/M2 phenotype, depending on the time as well as stage of disease in numerous GBM models (Figure 2) [9, 98, 107]. Hattermann et al. detected that GAMs from GBM patients secreted both M1 and M2 markers concurrently when freshly isolated GAMs from human GBM patients were compared to M1- and M2-polarized human macrophages [98]. Szulzewsky et al. established that GAMs from both murine glioma models exhibited gene secretion pattern that partially overlapped with specific M1 and M2 subsets (Figures 2) [9]. It was further revealed that GAM secretory profiles were very distinctive in glioma-associated phenotype, independent of conventional macrophage subsets [9]. The secretion of CD45 is the most frequently used method to differentiate resident microglia from infiltrating GAMs from the periphery. Microglia often exhibit mid to low CD45 secretion, while GAMs exhibit high CD45 secretion [4, 7].

GAMs were capable of augmenting the production of anti-inflammatory molecules like TGF-*β*1, arginase 1 (ARG1), and IL-10 which are associated with alternative macrophage activation and molecules like vascular endothelial growth factor (VEGF), matrix metalloproteinase (MMP)-2, MMP9, and MT1-MMP which supports tissue remodeling and angiogenesis (Figure 2) [8, 9]. Nevertheless, GAMs were capable of producing proinflammatory molecules like TNF*α*, IL-1*β*, and CXCL10 (Figure 2) [6, 8, 108]. Furthermore, GAMs were capable of augmenting the secretion several genes like Vegfa and Hgf which implicated in angiogenesis, ARG1 and Tgfb3 which are implicated in immune suppression, and MMP2, MMP14, and Ctgf which are implicated in tumor invasion (Figure 2) [9]. Thus, GAMs promote tumor growth instead of inhibiting tumor growth via the expression factors that support glioma invasion or immunosuppressive factors [6, 108].

Szulzewsky et al. demonstrated that genes like glycoprotein nonmetastatic b (GPNMB) and secreted phosphoprotein 1 (SPP1) were highly secreted by GAMs in different glioma mouse models as well as human GBM (Figure 2) [9]. They indicated that high secretion of these genes correlated well with shorter prognosis in patients with glioma [9]. They also established that GL261 cells secrete GPNMB at a very high concentrations in their experience involving GL261 glioma models [9]. Nevertheless, in their RCAS-PDGFb glioma mouse model, as well as in human GBM samples, GAMs were the major source of GPNMB secretion in all paired experimental samples [9].

Ripoll et al. demonstrated that GPNMB functions as a negative modulator of proinflammatory macrophage stimulated in RAW264.7 cells [109]. Therefore, high GPNMB secretion in GAMs was capable of inducing protumorigenic phenotypes of GAMs [109]. Studies have demonstrated that GPNMB was capable of blocking T cell stimulation via direct cell-cell intercommunication of antigen-presenting cells (APCs) and T cells resulting in immunosuppressive milieu in gliomas (Figure 2) [110, 111]. SPP1 was also recognized as a ligand for CD44, and SPP1-CD44 communication augmented the stemness of CD44 secretory glioma-stimulatory cells (Figure 2) [112]. Furthermore, it was established that GAMs and not any other cells in the tumor microenvironment were the principal source of SPP1 secretion in glioma [112].

## 8. Microglia and Macrophages in Glioma Invasion and Angiogenesis

Cancer invasion is a cell- and tissue-driven phenomenon via which the physical, cellular, and molecular factors adapt as well as respond during the entire development of the disease [113]. Cancer invasion is induced and sustained via signaling pathways that regulates cytoskeletal dynamics in tumor cells as well as the turnover of cell-matrix and cell-cell junctions, subsequent to cell migration into the neighboring tissue [113]. Angiogenesis is a regular physiological process, essential for normal tissue repair and growth [114]. In pathological situations, angiogenesis is often depicted by the unremitting proliferation of endothelial cells and blood vessel formation in [114]. Thus, angiogenesis is very fundamental in tumor growth, invasion, and metastasis [114].

**Figure 3:**
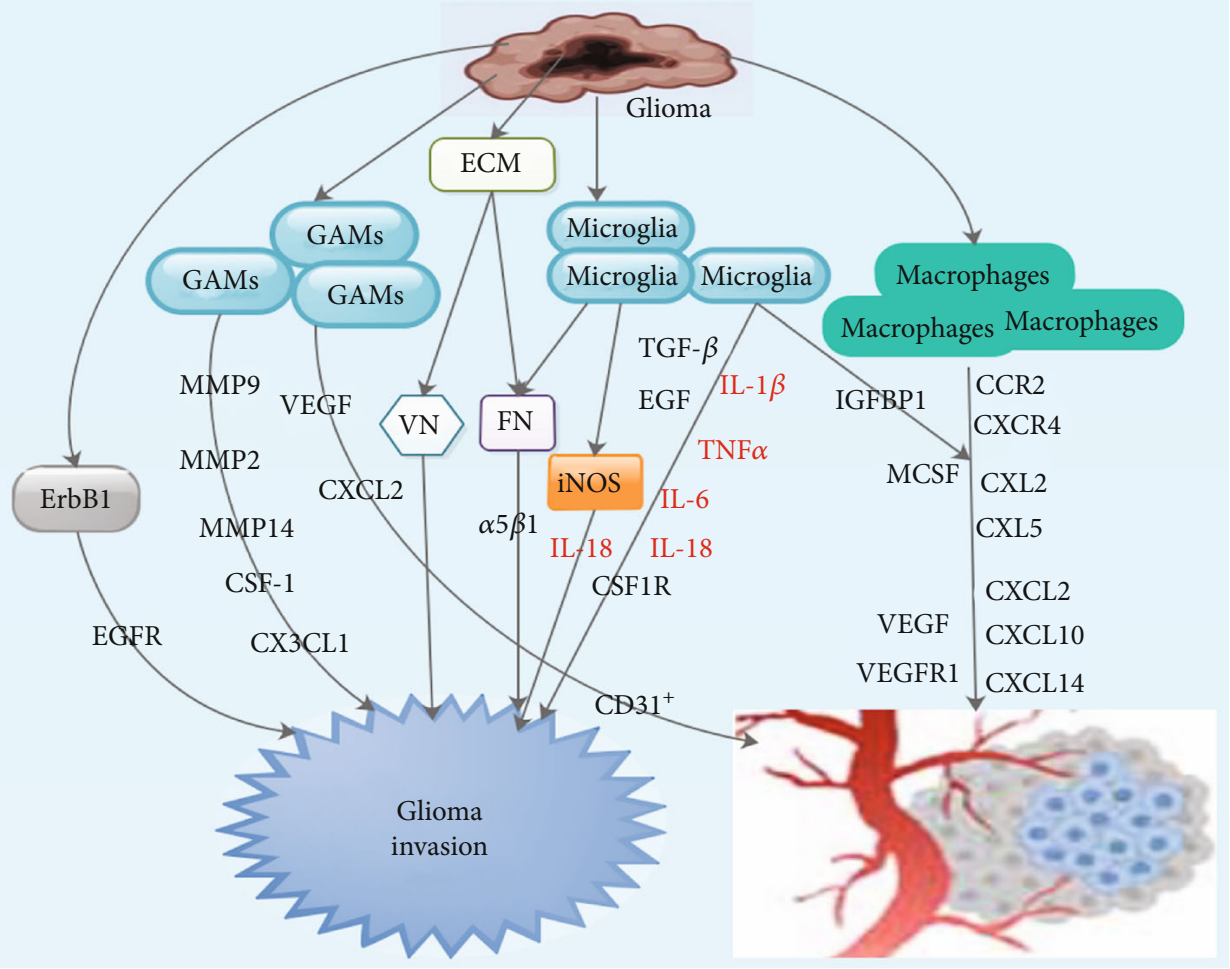
The influence of microglia, macrophages, and glioma-associated microglia and/or macrophages (GAMs) during glioma angiogenesis. Red factors = inhibitor/inhibition; black factors = facilitator/facilitation.

Yeh et al. demonstrated that matrix expressed precisely by glioma cells was able to condition microglia to express proinvasive factors [115]. They indicated that rat C6 astrocytoma cell line secreted excessive concentrations of extracellular matrix (ECM) proteins like fibronectin (FN) as well as vitronectin (VN), both of which are either nonexistent or secreted in extremely low concentrations in normal astrocytes (Figure 3) [115]. Färber et al. demonstrated that *α*5*β*1 was secreted by both glioma cells and microglia which indicates that influencing fibronectin signaling may influence both cell compartments (Figure 3) [116]. TGF-*β* is key growth factors that was capable of mediating microglia stimulation of glioma invasion (Figure 3) [117]. Coniglio et al. demonstrated that microglia were capable of secreting epidermal growth factor (EGF), most possibly the natural full-length precursor, on their surface [117, 118].

Studies have shown that ErbB1 was augmented in approximately half of human glioma samples [119–121]. Studies have further demonstrated that glioma cells are capable of interacting with epidermal growth factor receptor (EGFR) in an autocrine manner by cosecreting ErbB1 ligands (Figure 3) [122, 123]. Also, studies have shown that agents which were capable of inhibiting IL-1*β*, TNF*α*, and IL-6 secretion in microglia blocked their ability to induce GBM invasion (Figure 3) [124, 125]. Furthermore, inhibitory antibodies directed against IL-18 blocked BV-2 microglia from augmenting glioma cell migration as well invasion [117]. Nevertheless, recombinant IL-18 was capable of promoting C6 astrocytoma migration via stimulation of iNOS pathway (Figure 3) [117]. Studies have demonstrated that GAMs are capable of promoting GBM invasion via the modulation of MMPs (Figure 3) [117].

Ye et al. established that prime human glioma stem cells cocultured with GAMs prior to orthotopic grafting in NOD-SCID mice were more invasive compared to naïve glioma stem cells, and this correlated with upregulation of MMP9 in the tumor cells [5]. Held-Feindt et al. also demonstrated that human GAMs upregulated MMP2 as well as MMP9 mRNA secretion in response to exogenously administered CX3CL1 *in vitro* [126]. Moreover, Markovic et al. established that silencing of MMP14 in GAMs reduced GL261 tumor size [108]. Several studies have demonstrated that CSF1R was an invasion-related molecule responsible for normal microglial function (Figure 3) [127–129]. Furthermore, its ligand, CSF-1, was also implicated to augment GAM density (Figure 3) [130].

Brandenburg et al. revealed that, at the mRNA level, GAMs isolated from GL261 gliomas oversecreted proangiogenic molecules like VEGF and CXCL2 (Figure 3) [131]. They indicated that depletion of resident microglia explicitly led to a decrease in tumoral vessel counts analogous to that detected in total myeloid cell ablation, signifying that microglia had a more prominent role in angiogenesis compared to monocyte derived macrophages [131]. Furthermore, another study revealed that several GAMs associated with the tumor were detected in the perivascular niche in direct contact with CD31^+^ vessels which does not occur in the normal vasculature of nondiseased brain tissue (Figure 3) [132]. Moreover, it was observed that microglia often traffic through tumors along the vasculature in study involving microglia and the aberrant vasculature [132].

Bayerl et al. observed that both microglia and peripheral macrophages had direct interactions with vessels both in the periphery of and within the tumor in study involving allografted mice [132]. Also, significantly elevated levels of angiogenesis inducing factors like CCR2, CXCR4, CCL2, CCL5, CXCL2, CXCL10, CXCL14, VEGF, and VEGFR1 and a few other transcripts were detected in microglia as well as macrophages isolated from tumor-bearing animals (Figure 3) [133]. Nijaguna et al. revealed that glioma-secreted MCSF triggered angiogenesis *in vitro* as well as *in vivo* through macrophage-/microglia-secreted factors [134]. Also, it was established that recombinant human MCSF triggered angiogenesis through macrophages by facilitating VEGFA secretion [135].

Nijaguna et al. further established that MCSF present in the glioma cell conditioned medium influenced monocytes or microglial cells as well as facilitated the expression of factors, which triggered angiogenesis *in vitro* (Figure 3) [134]. They indicated that angiogenesis triggered by monocyte/microglia exposed to glioma cell conditioned medium was dependent on the stimulation of MCSF/MCSFR signaling in macrophages [134]. Furthermore, a search of novel mediators of angiogenesis in the microglial secretome by means of an unbiased proteomic strategy detected 67 proteins expressively modulated by MCSF [134]. Moreover, it was observed that VEGFA level was augmented in microglial cell secretomes through a MCSF-dependent mechanism [134].

Nijaguna et al. further established that glioma-derived MCSF upregulated IGFBP1 level in the secretome of microglial cells (Figure 3) [134]. Also, neutralizing IGFBP1 by a specific antibody in the microglial secretome after the administration of glioma cell conditioned medium spicily decreased tube formation in HUVEC, implying that stimulation of MCSF which resulted in upregulation of IGFBP1 secretion by microglial cells was fundamental for angiogenesis [134]. Thus, IGFBP1 expressed by microglial cells in response to glioma-expressed MCSF was perhaps the main origin of tumor angiogenesis (Figure 3) [134].

## 9. Microglia and Macrophages Signing Pathways in Glioma

**Figure 4:**
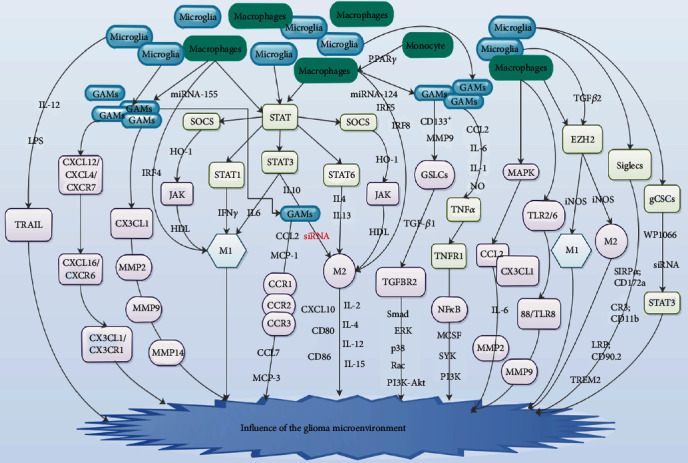
Signaling pathways of microglia, macrophages, and glioma-associated microglia and/or macrophages (GAMs) at the glioma microenvironment. Red factors = inhibitor/inhibition; black factors = facilitator/facilitation.

Several pathways through microglia and macrophages that intercommunicate with other tumor development factors have been described in glioma [71, 136–138]. Microglia and macrophages utilize a variety of receptors for the detection of molecules, particles, and cells that they engulf [15, 139]. Several members of the signal transducer and activator of transcription (STAT) family, such as STAT1, STAT3, and STAT6, have been implicated in phenotypic switching of microglia as well as macrophages (Figure 4) [71, 136]. Several studies have demonstrated that STAT1 triggered M1 polarization after introducing IFN­*γ*, whereas STAT6 was key in forming M2 phenotype after stimulating IL­4 as well as IL­13 (Figure 4) [140–142]. Furthermore, STAT3 was capable of triggering both IL­10­activated M2 polarization and IL­6­activated M1 polarization (Figure 4) [143, 144].

McFarland et al. established that STAT3 was capable of modulating M1 antitumor response [7]. Moreover, it was further established that STAT3 was capable of stimulating GAMs which was responsible for M2 phenotype [7]. It was further demonstrated that augmented GAM STAT3 stimulation resulted in a reduction of M2 GAM infiltration in tumors of SOCS3^fl/fl^ mice compared to SOCS3^−/−^ mice *in vivo* [7]. Studies revealed that siRNA blockade of STAT3 in glioma cells resulted in microglial stimulation as well as tumor growth blockade in murine models, with upsurges in IL-2, IL-4, IL-12, IL-15, and CXCL10 as well as upregulation of CD80 and CD86 on myeloid cells (Figure 4) [137, 138]. Studies have further demonstrated that cytokines like heme oxygenase 1 (HO-1) and HDL were capable of modulating macrophage phenotypes via the SOCS-JAK-STAT pathway (Figure 4) [145, 146].

Studies have demonstrated that GAMs were recruited to sites of gliomas via interaction between CCL2/MCP-1 which are basically generated by glioma cells as well as its receptor CCR2 which is secreted by GAM and by a CCL7/MCP-3-CCR1/CCR2/CCR3-crosstalk (Figure 4) [98, 147–149]. Furthermore, small chemotactic cytokines, known as chemokines, are fundamental participants in GBM progression because they are capable of accelerating the infiltration of GAMs into glioma tissues [98, 147–149]. Held-Feindt et al. demonstrated that CX3CL1 stimulated recruitment of GAMs into GBM as well as augmented the secretion of the MMP 2, 9, and 14 in the tumor cells (Figure 4) [126]. Studies further exhibited that chemokine/receptor pairs like CXCL12/CXCR4/CXCR7, CXCL16/CXCR6, and CX3CL1/CX3CR1 were obviously associated with tumor progression (Figure 4) [126, 150, 151].

Hattermann et al. revealed that GAMs are depicted with the secretion of these chemokine/receptor pairs signifying an essential function of this secretory profile in GAM biology in gliomas [98]. Bouhlel et al. established that PPAR*γ* was capable of facilitating monocyte differentiation into macrophages primed toward M2 polarization, which had anti-inflammatory properties (Figure 4) [152]. Studies have demonstrated that IRFs exercise different functions in macrophage polarization. It was observed that IRF4 was a fundamental transcription factor regulating M2 polarization, while IRF­5 and IRF­8 modulated M1 polarization (Figure 4) [153, 154]. Studies have further demonstrated that M1 and M2 phenotypes correlated well with distinct miRNA profiles [71, 155].

Studies revealed that miRNA­155 secretion stimulated M1 polarization via a knockdown of mRNAs transcription from M2 signature genes, while miRNA­124 stimulated M2 polarization by influencing mRNA transcription from M1 genes [156, 157]. Bruna et al. established that robust action of TGF-*β* signaling pathway in human glioma tissues correlated with poor prognosis [158]. Studies have demonstrated that infiltrating leukocytes were responsible for the buildup of TGF-*β*1 at the invasive front portion of tumor, while glioma cells were capable of producing TGF-*β*2 [158–161]. Studies have further showed that TGF-*β* binds to type II TGF-*β* receptor- (TGFBR-) 2 once stimulated [158–161].

Studies further established that the ligand-bound TGFBR2 was capable of efficiently transstimulating the TGFBR1, which transduced intracellular signals via canonical Smad-dependent and/or Smad-independent pathways like ERK, p38, Rac, and PI3K-Akt pathways (Figure 4) [159, 160, 162]. Wesolowska et al. revealed that inhibition of TGFBR2 was capable of blocking the invasion of glioma cells [163]. Studies have also demonstrated that TGF-*β* was capable of influencing cancer development processes like cell invasion, immune suppression, and microenvironment modification [159, 160]. Ye et al. demonstrated that GAMs expressively augmented the invasive ability of glioma stem-like cells (GSLCs) via paracrine generation of TGF-*β*1 as well as the TGF-*β*1-TGFBR2 signaling pathway (Figure 4) [5].

Ye et al. observed that GAMs were profoundly disseminated at the invasive front region of glioma and was associated with CD133^+^ glioma cells like GSLCs and these GAMs generated robust quantities of TGF-*β*1 (Figure 4) [5]. They indicated further that augmented invasive potential of GSLCs was triggered by elevated generation of MMP9 from CD133^+^ GSLCs by paracrine TGF-*β*1 from GAMs via TGFBR2 pathway (Figure 4) [5]. Studies have showed that glioma-infiltrating GAMs were polarized to M2 phenotype by secreting robust quantities of immunosuppressive cytokines like IL-10 and TGF-*β*1 which were capable of augmenting tumor immune suppression as well as accelerated tumor progression [164–166].

Studies have also demonstrated that TNF*α* was capable of triggering the expression of CCL2, IL-6, IL-1, and NO which are expressively generated by GAMs (Figure 4) [107, 167]. Studies have shown that TNF receptor 1 (TNFR1) stimulation was capable of degrading I*κ*Ba, a blocker of NF*κ*B signaling. It was further observed that this degradation triggered a positive feedback loop with p65/p50 nuclear translocation resulting in the transcriptional stimulation of TNF*α* [168, 169]. Also, NF*κ*B stimulation triggered promigratory genes that participated in tumor invasiveness associated with numerous protumor chemokines as well as the MMP pathways [170]. TNFR1 and the related NF*κ*B pathway in microglia could be involved in glioma pathogenesis (Figure 4) [168]. Thus, further studies are needed in this direction.

Cytokines exhibiting an augmented level in GBM serum comprised of MCSF, which was also upregulated in GBM tissue via a mechanism dependent on the SYK-PI3K-NF*κ*B pathway (Figure 4) [134]. Chiu et al. established that administration of IL-12 as well as LPS discernibly augmented microglial phagocytotic activity via the TRAIL pathway (Figure 4) [171]. Studies revealed that GAMs exhibited protumorigenic properties by upregulation of MMP-2 but did not express cytokines like IL-1*β*, IL-6, and TNF*α*, which are distinctive from the inflammatory phenotype (Figure 4) [80, 172]. A study revealed that the significance of IL-1*β* lies in the IL-1*β*/CCL2/IL-6 interaction between microglia as well as glioma cells (Figure 4) [173].

Also, a study revealed that IL-1*β* secreted by both microglia and macrophages stimulated the p38 mitogen-activated protein kinase (MAPK) pathway in glioma cells, which in turn results in augmented secretion of CCL2—the agonist for CCR2 on microglia (Figure 4) [173]. This led to an upsurge in microglial production of IL-6 and subsequently MMP2, which accelerated tumor migration, invasion, and gliomagenesis [174, 175]. The CX3CR1/IL-1*β*/CCL2 pathway was capable of decreasing IL-6 protumor signaling as well as the blockade of MMP-based pathways in microglia (Figure 4) [173]. GBM cells triggered TLR2/6 stimulation in both macrophages and microglia via the myeloid differentiation primary response 88/TLR8 signaling pathway, which in turn results in the augmentation of MMP-9 which accelerate tumor invasion as well as angiogenesis (Figure 4) [78, 176].

Enhancer of zeste homolog 2 (EZH2) is the core catalytic subunit of Polycomb repressive complexes 2 (PRC2) [1, 177]. Yin et al. demonstrated that EZH2 suppression in GBM triggered polarization shift of microglia as well as PMMC-derived macrophage resulting in the augmentation of M1 markers as well as decrease in M2 markers [1]. Yin et al. indicated that EZH2 blockade in GBM reduced the secretion of M2 markers as well as augmented the secretion of M1 markers in coculturing microglia and that iNOS was associated with EZH2-mediated microglial polarization shift (Figure 4) [1]. They further indicated that EZH2 blockade in GBM resulted in decreased TGF*β*1 as well as TGF*β*2, whereas TGF*β*2 promoted microglia capabilities (Figure 4) [1]. Thus, TGF*β*2 was associated with EZH2-mediated GBM progression as well as growth [1].

In glioma milieu, normal human astrocytes, glioma cells, and microglia all exhibited the ability of phagocytosing glioma cells and precisely apoptotic tumor cells [178, 179]. Sialic acid binding immunoglobulin-like lectins (Siglecs) are significant modulatory receptors secreted by microglia and bind to sialated ligands on neurons or CNS tumor cells [180–182]. Siglec signaling modulated the stimulation of microglia as well as phagocytosis activity [180–182]. Studies established that they also serve as binding receptors which triggered signaling via signal-regulatory protein alpha (SIRP*α* and CD172a), complement receptor 3 (CR3 and CD11b), low-density lipoprotein receptor-related protein (LRP and CD90.2), and protein triggering receptor expressed on myeloid cells-2 (TREM2) (Figure 4) [15, 183–185].

Studies have further demonstrated that Siglecs are capable of regulating phagocytosis via microglia, specifying that live neurons are capable of regulating phagocytosis via the secretion of corresponding ligands [183–185]. Wu et al. showed that glioma cancer stem cells (gCSCs) were capable of inhibiting phagocytosis via human microglia *in vitro* [165]. Furthermore, reversal of phagocytosis inhibition after STAT3 silencing by WP1066 as well as STAT3 siRNA in gCSCs resulted in the inhibition of phagocytosis (Figure 4) [165, 178]. This suggested that pSTAT3 pathway was capable of inhibiting phagocytosis via gCSCs [165].

## 10. Microglia and Macrophages in Glioma Therapy

**Figure 5:**
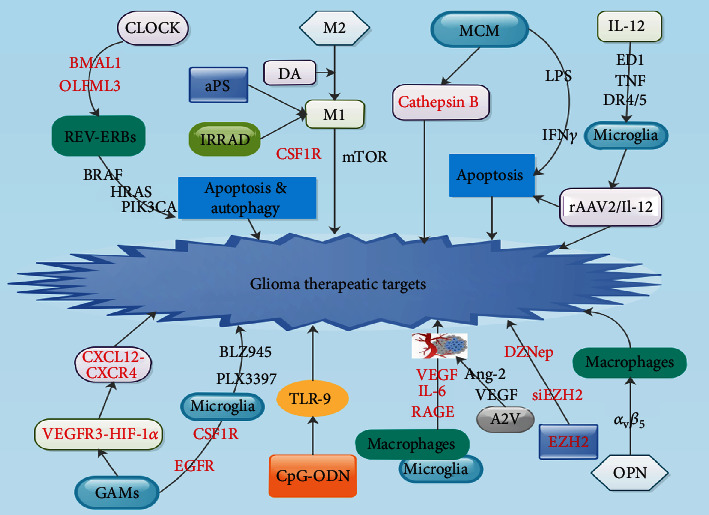
Therapeutic potentials of microglia, macrophages, and glioma-associated microglia and/or macrophages (GAMs) in glioma. Red factors = inhibitor/inhibition; black factors = facilitator/facilitation.

Studies have shown that the abundance of GAMs positively correlated with GBM invasiveness, immunosuppression, and patients' poor outcome, making these cells a suitable target for immunotherapeutic schemes [91, 186, 187]. The conversion of M2 macrophages to M1 macrophages has been implicated as a potential treatment strategy to reduce glioma growth [94]. Proinflammatory M1 macrophages have demonstrated to be of therapeutic target in cancer due to their antitumor functional properties (Figure 5) [7]. Zeiner et al. observed that an M1-polarized immune microenvironment correlated with prolonged survival in patients with GBM [188]. Studies have demonstrated that introduction of amphotericin or CSF1R blockade stimulated M1 activity in GBM (Figure 5 and Table 1) [189, 190].

Lisi et al. observed that mTOR kinase inhibitor stimulation in glioma triggered microglia to secrete M1 phenotypes (Table 1) [191]. Furthermore, introduction dopamine (DA) blocked tumor growth by reprogramming M2-polarized macrophages to M1 phenotypes in a rat model of glioma (Table 1) [192].

Studies have shown that agents like low dose irradiation (IRRAD) as well as phosphatidylserine antibody (aPS) administration triggered a proinflammatory M1 phenotype in tumor-associated microphages that reduced tumor growth in other cancer models (Figure 5 and Table 1) [193, 194]. Studies with these agents are warranted in glioma. Chen et al. discovered that OLFML3 is a novel as well as potent CLOCK-modulated microglia chemoattractant in GBM. They demonstrated that OLFML3 depletion augmented survival in patients with GBM [10].

It was established that the fundamental function of the CLOCK : BMAL1 complex in GBM tumor biology, specifically its modulation of precise metabolic as well as immunity genes like OLFML3, elucidates hypothetical therapeutic targets governing fundamental cancer hallmarks of stemness as well as immune suppression [10]. Furthermore, pharmacological stimulation of circadian clock components REV-ERBs, which repress transcription of CLOCK as well as BMAL1, was capable of inhibiting the growth of GBM (Figure 5 and Table 1) [10, 195]. Precisely, stimulation of REV-ERBs was selectively toxic to glioma cells by influencing oncogenic drivers such as HRAS, BRAF, and PIK3CA which triggered apoptosis as well as blocked autophagy (Figure 5 and Table 1) [10, 195].

Hwang et al. established that microglia conditioned culture medium (MCM) facilitated apoptosis of glioma cells, with extra cytotoxic effect when microglial cells were introduced to LPS or IFN*γ in vitro* study (Figure 5 and Table 1) [196]. They observed that this influence was glioma-specific, without unwanted astrocyte cytotoxicity. Furthermore, proteonomic evaluation of the MCM revealed LPS- and IFN*γ*-associated proteins together with significantly elevated secretion of cathepsin proteases such as cathepsin B (Figure 5 and Table 1) [196]. Also, glioma apoptosis was no longer detected when cathepsin B was suppressed, signifying this protein's importance in microglial antitumor function [196].

Preclinical trials demonstrated that local administration of oligodeoxynucleotides containing CpG motifs (CpG-ODN) had robust immunostimulatory effects as well as activation of toll-like receptor- (TLR-) 9 in both microglia and macrophages (Figure 5 and Table 1) [197]. Carpentier et al. revealed that the use of CpG-ODN in a murine *in vivo* glioma model triggered a reduction in tumor size without toxicity to brain parenchyma (Table 1) [198]. Chiu et al. established that IL-12 was capable of activating microglia because it was able to augment the secretion of ED1 as well as TNF-associated apoptosis-stimulating ligand using recombinant adenovirus-carrying IL-12 (rAAV2/IL-12) (Figure 5) [199]. Studies have further demonstrated that IL-12 treatment was capable of triggering microglial-mediated apoptosis of GBM cells via DR4/5 binding *in vitro* (Figure 5 and Table 1) [171, 199].

Tabouret et al. indicated that, with tumor recurrence, there was a switch in secretory profile from VEGFR3-HIF-1*α* to CXCL12-CXCR4 predominance in GAMs (Figure 5) [200]. Thus, microglia may have roles in propagating extra mechanisms of immune resistance in tumor recurrence, offering alternative basis for scrutinizing as well as targeting this population to augmented antitumor strategies [200]. Studies have demonstrated that GAMs secreted EGF as well as CSF1R, whereas GBM cells secreted EGFR as well as CSF-1 to create a paracrine loop [118, 201]. Coniglio et al. revealed that blockade of EGFR as well as CSF1R in cocultures of murine microglia/macrophages and GL261 cells inhibited GAM augmented of invasiveness (Figure 5 and Table 1) [118].

Coniglio et al. further demonstrated that introduction of PLX3397, a CSF-1R inhibitor, was capable of decreasing the recruitment of GL261-associated microglia/macrophages as well as glioma invasiveness *in vivo* (Figure 5 and Table 1) [118]. Pyonteck et al. also demonstrated that the use of CSF1R inhibitor, BLZ945, inhibited the progression of intracranial xenografts of conventional human glioma cells via the facilitation of GAM antitumor gene secretion (Figure 5 and Table 1) [190]. Chen et al. demonstrated that IL-6 and VEGF secretion were inhibited in GL261-associated microglia/macrophages after silencing receptor for advanced glycation end products (RAGE) as was angiogenesis (Figure 5 and Table 1) [202]. Introduction of A2V, a bispecific antibody to VEGF, and Ang-2, another proangiogenic factor, was capable of overcoming resistance to therapies directed against the VEGF pathway in both the GL261 and human glioma stem cell xenograft mouse models (Figure 5 and Table 1) [127].

Margueron and Reinberg demonstrated that EZH2 was capable of silencing a bundle of tumor suppressor genes via methylation of lysine 27 of histone 3 (H3K27) of target genes [177]. Studies further demonstrated that EZH2 was extremely abundant in GBM samples and that secretory levels of EZH2 positively correlated with GBM grades as well as unfavorable survival [203, 204]. Thus, EZH2 could be a diagnosis as wells prognosis biomarker for glioma. Studies established that silencing of EZH2 by siEZH2 or function suppression by DZNep triggered GBM growth inhibition (Figure 5) [205, 206]. Furthermore, EZH2 was capable of modulating several tumor processes like cell cycle, proliferation, apoptosis, invasion, and mobility, GBM stem cell differentiation and maintenance, and tumor angiogenesis [1, 205]. Studies on EZH2 and microglia/microphages or GAM axis are needed to evaluate their influence in glioma.

Wei et al. revealed that osteopontin (OPN) had substantial predictive potential in determining survival of patients with GBM [207]. Also, OPN was associated with the mesenchymal subtype—known to be augmented with polarized macrophages [207]. Furthermore, it was observed that OPN secretory levels directly correlated with several macrophage markers in GBM specimens. Moreover, OPN was upregulated in the tumor milieu via the OPN/integrin *α*_v_*β*_5_ pathway [207]. OPN-mediated chemokine properties of macrophages were based on the intercommunication between OPN and integrin *α*_v_*β*_5_ (Figure 5 and Table 1) [207]. A study established that OPN^–/–^ microenvironment augmented survival in mice bearing intracerebral GL261 tumors [207]. Nevertheless, another study did not find any significant augmentation in survival [9].

## 11. Conclusion

Microglia are the precise phagocytes of the CNS, whereas macrophages are myeloid immune cells that are depicted with fervent phagocytosis. Macrophages are derived from circulating monocytes because, in pathological conditions, monocytes produce macrophages. Microglia and macrophages utilize a variety of receptors for the detection of molecules, particles, and cells that they engulf. In glioma milieu, normal human astrocytes, glioma cells, and microglia all exhibited the ability of phagocytosing glioma cells and precisely apoptotic tumor cells. GAMs positively correlated with GBM invasiveness, immunosuppression, and patients' poor outcome, making these cells a suitable target for immunotherapeutic schemes.

## Figures and Tables

**Table 1:** A summary of drugs or chemical agents related to glioma-associated microglia and/or macrophages targets.

Drug/chemical agent	Influence on microglia and macrophages in glioma therapy	Source/reference
Amphotericin	Stimulation of M1 activity in GBM	[189, 190]
CSF1R blockade	Stimulation of M1 activity in GBM	[189, 190]
mTOR kinase inhibitors	Microglia to secretion of M1 phenotypes in GBM	[191]
Dopamine	Reprogramming of M2-polarized macrophages to M1 phenotypes in glioma	[192]
Irradiation	Stimulation of proinflammatory M1 phenotype in cancer models	[193, 194]
Phosphatidylserine antibody	Stimulation of proinflammatory M1 phenotype in cancer models	[193, 194]
OLFML3	Potent CLOCK-modulated microglia chemoattractant in GBM	[10]
REV-ERBs	Repress transcription of CLOCK as well as BMAL1 was capable of inhibiting the growth of GBM Selectively toxic to glioma cells by influencing oncogenic drivers such as HRAS, BRAF, and PIK3CA which triggered apoptosis as well as blocked autophagy	[10, 195]
LPS or IFN*γ*	Facilitation of apoptosis of glioma cells, with extra cytotoxic effect	[196]
Oligodeoxynucleotides (CpG-ODN)	Robust immunostimulatory effects as well as activation of TLR-9 in both microglia and macrophages	[197]
CpG-ODN	Reduction in tumor size without toxicity to brain parenchyma	[198]
IL-12	Microglial-mediated apoptosis of GBM cells via DR4/5 binding *in vitro*	[171, 199]
EGFR and CSF1R blockade	Inhibition of GAM augmented invasiveness of glioma	[118]
PLX3397	Decreased the recruitment of GL261-associated microglia/macrophages as well as glioma invasiveness *in vivo*	[118]
BLZ945	Inhibition of the progression of intracranial xenografts of conventional human glioma cells via the facilitation of GAM antitumor gene secretion	[190]
Advanced glycation end products (RAGE)	Inhibition of IL-6, VEGF secretion, and angiogenesis in GL261-associated microglia/macrophages	[202]
A2V	Overcoming resistance to therapies directed against the VEGF pathway in both the GL261 as well as human glioma stem cell xenograft mouse models	[127]
Osteopontin (OPN)	OPN-mediated chemokine properties of macrophages were based on the intercommunication between OPN and integrin *α*_v_*β*_5_	[207]

## Data Availability

No data was used in this paper

## Abbreviations

ARNTL: Aryl hydrocarbon receptor nuclear translocator-like protein 1
P2Y: Adenosine diphosphate receptor
ATP/ADP: Adenine nucleotides
ARG1: Arginase 1
APCs: Antigen-presenting cells
BMAL1: Brain and muscle ARNT-like 1
BM: Bone marrow
CLOCK: Circadian locomotor output cycles kaput
CRY: Cryptochrome
CNS: Central nervous system
CSF1: Colony stimulating factor 1
CSF1R: CSF1 receptor
DAMPs: Damage-associated molecular patterns
DAP12: DNAX activation protein of 12 kDa
ECM: Extracellular matrix
EZH2: Enhancer of zeste homolog 2
EGF: Epidermal growth factor
CX3CR1: Fractalkine receptor
GAMs: Glioma-associated microglia and/or macrophages
GLUT: Glucose transporter
GBM: Glioblastoma multiforme
GPNMB: Glycoprotein nonmetastatic b
GSLCs: Glioma stem-like cells
gCSCs: Glioma cancer stem cells
IL: Interleukin
IRF: Interferon regulatory factor
IFN: Interferon
Iba1: Ionized calcium-binding adaptor molecule
LPS: Lipopolysaccharides
LRP: Lipoprotein receptor-related protein
M-CSF: Macrophage-colony stimulating factor
MCP-1: Monocyte chemoattractant protein
MAPK: Mitogen-activated protein kinase
MHC: Major histocompatibility complex
MMP: Matrix metalloproteinase
H3K27: Methylation of lysine 27 of histone 3
iNOS: Nitric-oxide species
TNFR1: TNF receptor 1
PER: Period
PRRs: Pattern recognition receptors
PAMPs: Pathogen-associated molecular patterns
PRC2: Polycomb repressive complexes 2
CpG-ODN: Oligodeoxynucleotides containing CpG motifs
OPN: Osteopontin
rAAV2/IL-12: Recombinant adenovirus-carrying IL-12
RAGE: Receptor for advanced glycation end products
SDF-1: Stromal-derived factor-1
SAMPs: Self-associated molecular patterns
SPP1: Secreted phosphoprotein 1
STAT: Signal transducer and activator of transcription
Siglecs: Sialic acid binding immunoglobulin-like lectins
SIRP*α*: Signal-regulatory protein alpha
TNF*α*: Tumor necrosis factor alpha
Th: T helper
Treg: T regulatory
TGF-*β*: Transforming growth factor-beta
TGFBR: TGF-*β* receptor
TREM2: Triggering receptor expressed on myeloid cells-2
TLR: Toll-like receptor
VEGF: Vascular endothelial growth factor.
